# High-Silica Zeolites as Sorbent Media for Adsorption and Pre-Concentration of Pharmaceuticals in Aqueous Solutions

**DOI:** 10.3390/molecules25153331

**Published:** 2020-07-22

**Authors:** Elena Sarti, Tatiana Chenet, Claudia Stevanin, Valentina Costa, Alberto Cavazzini, Martina Catani, Annalisa Martucci, Nicola Precisvalle, Giada Beltrami, Luisa Pasti

**Affiliations:** 1 Department of Chemical and Pharmaceutical Sciences, University of Ferrara, Via Luigi Borsari 46, 44121 Ferrara, Italy; tatiana.chenet@unife.it (T.C.); claudia.stevanin@unife.it (C.S.); valentina.costa@unife.it (V.C.); alberto.cavazzini@unife.it (A.C.); martina.catani@unife.it (M.C.); luisa.pasti@unife.it (L.P.); 2 Department of Physics and Earth Sciences, University of Ferrara, Via Giuseppe Saragat 1, 44122 Ferrara, Italy; annalisa.martucci@unife.it (A.M.); nicola.precisvalle@unife.it (N.P.); giada.beltrami@unife.it (G.B.)

**Keywords:** adsorption, emerging contaminants, pre-concentration, sorbent regeneration, zeolites

## Abstract

The present work focused on the use of high-silica commercial zeolites as sorbent media for pharmaceuticals in an aqueous matrix. As drug probes, ketoprofen, hydrochlorothiazide, and atenolol were selected, because of their occurrence in surface waters and effluents from wastewater treatment plants. Pharmaceuticals adsorption was evaluated for two Faujasite topology zeolites with Silica/Alumina Ratio 30 and 200. The selected zeolites were demonstrated to be efficient sorbents towards all investigated pharmaceuticals, thanks to their high saturation capacities (from 12 to 32% w/w) and binding constants. These results were corroborated by thermal and structural analyses, which revealed that adsorption occurred inside zeolite’s porosities, causing lattice modifications. Finally, zeolites have been tested as a pre-concentration media in the dispersive-solid phase extraction procedure. Recoveries higher than 95% were gained for ketoprofen and hydrochlorothiazide and approximately 85% for atenolol, at conditions that promoted the dissolution of the neutral solute into a phase mainly organic. The results were obtained by using a short contact time (5 min) and reduced volume of extraction (500 µL), without halogenated solvents. These appealing features make the proposed procedure a cost and time saving method for sample enrichment as well as for the regeneration of exhausted sorbent, rather than the more energetically expensive thermal treatment.

## 1. Introduction

The increasing use of pharmaceutical compounds in human and veterinary medicine has become an environmental problem, which received widespread attention from scientists over the past 20 years [1,2]. Hundreds of tonnes of pharmacologically active substances enter sewage treatment plants, where they are poorly removed. Consequently, they can enter into surface water collecting the plant effluents [3,4]. Indeed, the occurrence of pharmaceuticals in surface water and sediments has been extensively reviewed [5,6]. Likewise, “source-to-tap” studies concerning the fate of pharmaceuticals from wastewater to finished drinking water have been reported [7,8]. The concentrations measured in natural and drinking waters are very low, generally in the ng L^−1^ to μg L^−1^ [9,10], thus requiring accurate and sensitive methods for their detection. Likewise, to reduce the negative impact of pharmaceutical on biota and human health, new technologies should be investigated to improve the efficiency of water treatment plants in the removal of drugs. Therefore, it is important on one hand to develop analytical methods able to detect these molecules at a low concentration level to better evaluate their impact on the environment and, on the other hand, to enhance the efficiency of water remediation technologies in order to reduce water contamination. Sorption based technologies were demonstrated to be efficient and economical methods both for the enrichment of trace pollutants as well as for their removal from water [11,12,13]. Among the wide variety of adsorbents, zeolites are very promising for environmental application thanks to their properties such as high surface area, controlled pore size, thermal and chemical stability. Recent studies demonstrated the efficiency of zeolites in the adsorption of several organic pollutants from water, such as pharmaceuticals [14,15], polycyclic aromatic hydrocarbons [16,17], and petrol-derived compounds such as toluene [18], methyl tert-butyl ether [19], chlorobenzene [20]. In addition, zeolites were also investigated as materials for the enrichment of organic compounds in dilute aqueous solutions. Dispersive solid-phase extraction is a promising sample pre-treatment technique, first introduced by Anastassiades et al. [21], which is based on the SPE methodology, but the sorbent is dispersed in a sample solution containing the target analytes instead of being immobilized inside a cartridge. This approach enables the sorbate to interact equally with all the sorbent particles and avoids channeling or blocking of cartridges or disks, as occurs in traditional SPE [22]. Other advantages with respect to the conventional SPE are the small sample volume required, reduced operational time, and high recoveries [23]. The dispersive-SPE has been successfully introduced for several analyses (i.e., pesticide residues in various matrices, antibiotics in animal tissues, pharmaceuticals in whole blood), with the purpose of sample clean-up and elimination of matrix interferences [24]. In the present work, the adsorptive properties of high-silica zeolites toward drugs in aqueous solutions were studied in order to investigate the efficiency of these siliceous materials in two applications, namely: (1) The removal of contaminants from the aqueous matrix, (2) the pre-concentration step for micropollutants analysis. An interesting future perspective of the present work could be the investigation of the possible reuse of zeolites after solvent extraction of sorbed analytes. Despite the fact that the thermal treatment usually requires high temperatures and long times, it still represents the most common regeneration technique of an exhausted adsorbent. It has been already demonstrated that exhausted zeolites maintain their adsorption efficiency after thermal regeneration [25,26]. Adsorbent regeneration by solvent extraction could be an attractive alternative to thermal treatment, because it is time-saving, requires less energy, and could permit the recovery of bioactive molecules from waste. Target pharmaceuticals were chosen based on their presence in effluents of wastewaters treatment plants and in surface waters [3,27,28]. In detail, the three investigated pharmaceuticals were ketoprofen (non-steroidal anti-inflammatory drug), hydrochlorothiazide (diuretic), and atenolol (beta-blocker), which are characterized by different physicochemical properties. As adsorbent media, commercial high-silica zeolites [29] (FAU and BEA framework type), differing in structure and surface properties, were considered. The release of sorbed pharmaceuticals from zeolites was evaluated by varying the pH and organic modifier of the extracting phase.

## 2. Results and Discussion

### 2.1. Adsorption

The adsorption of pharmaceuticals from the aqueous solution onto zeolites was evaluated in a wide concentration range and by fitting the experimental data with the proper mathematical model. In this case, good fits were obtained using Langmuir adsorption isotherm Equation (1) which has been already employed to investigate several organic molecule-zeolite systems [25,30,31]:

(1) $$q=\frac{q_{S}bC_{e}}{1+bC_{e}}$$

**Figure 1:**
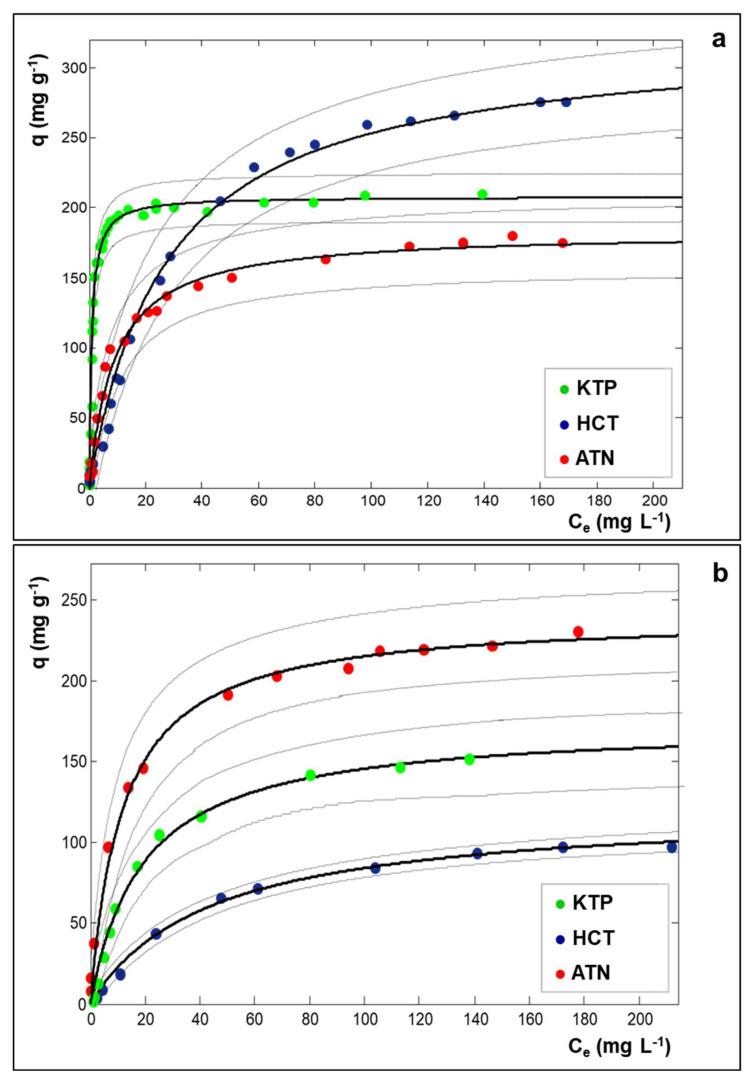
Adsorption isotherms of ketoprofen (KTP: green symbols), hydrochlorothiazide (HCT: dark blue symbols), and atenolol (ATN: red symbols) on Y200 (**a**) and Y30 (**b**) in Milli-Q water. Dotted lines are the confidence limits at 95% of probability of the fitted curves.

In Equation (1), q (mg g^−1^) is the adsorbed amount per unit weight of adsorbent, C_e_ (mg L^−1^) is the solute concentration at equilibrium, q_S_ is the saturation capacity (mg g^−1^) and b (L mg^−1^) is the Langmuir coefficient (binding constant). The adsorption isotherms of drugs adsorption on Y zeolites in Milli-Q water are shown in Figure 1 and the respective parameters estimated by non-linear fitting are reported in Table 1. Data concerning the drugs adsorption onto Beta25 are reported elsewhere [15].

**Figure 2:**
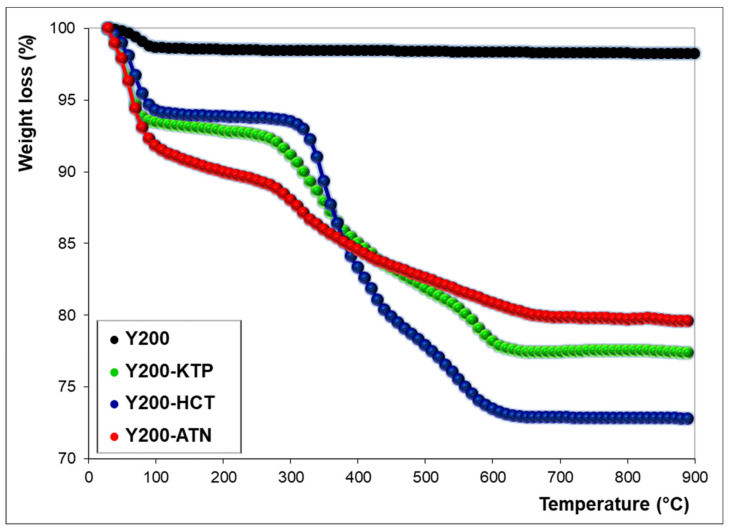
Thermogravimetric curves of Y200 before (black) and after drugs adsorption (KTP: Green, HCT: Blue, ATN: Red).

As can be seen in Table 1, both USY zeolites show high saturation capacities toward all the examined drugs. These data could be compared with saturation capacities approximately ranging from 20 to 100 mg g^−1^ found by other studies for the adsorption of pharmaceuticals by different adsorbents, such as carbon black [32], granular or derivatized activated carbon [33,34], polymer clay composite [35]. In particular, by comparing the adsorptions on Y200, it can be noted that the highest saturation capacity (q_S_) was reached for HCT, but the isotherm slope (b), related to the adsorption constant, is the lowest one. At low concentrations, Y200 shows the greatest adsorption efficiency towards KTP. From the data of Table 1, it can also be observed that Y30 shows higher adsorption capacity and affinity for ATN than for the other pharmaceuticals. This last finding could be ascribed to electrostatic interactions between Y30, which is less hydrophobic than Y200, and ATN which is partially positively charged. By comparing the USY zeolites in terms of saturation capacities and binding constants (Table 1), the more hydrophobic Y200 generally demonstrated to offer higher efficiency than Y30, despite the lower surface area (see Table S1), thus confirming that the surface area is not exhaustive for predicting adsorption properties. In the light of above, further investigations were performed solely on Y200. In order to obtain more information on the adsorption process, thermal and diffractometric analyses were carried on. Thermogravimetric curves of Y200 before and after saturation with drugs are shown in Figure 2.

The TG curves of as-synthesized Y200 show a total weight loss of 1.7% at 900 °C. The thermal profile shows a sudden slope variation at a temperature below 100 °C, thus suggesting the presence of water molecules weakly bonded to the surface [14]. The TG curves of Y200 saturated with drugs display two main weight losses: The first one below 100 °C can be attributed to the desorption of species (water and/or drug) weakly retained on the zeolite surface, while the second one at temperatures above 100 °C can be ascribed to the decomposition of drug sorbed inside the zeolite porosities. According to the literature data [36,37,38], KTP and HCT present a stable profile until about 250 and 300 °C, respectively, while the ATN thermal decomposition starts at lower temperatures (120–150 °C).

Thermogravimetric curves of Y200 after saturation with ATN, HCT, and KTP show weight losses at 900 °C of 20.4%, 27.2%, and 22.6%, respectively. These results indicated that, at saturation conditions, the higher amount of sorbed drug was found for HCT, followed by KTP and ATN. The same trend can be observed by comparing the saturation capacities extrapolated from adsorption isotherms (see q_S_ in Table 1). Differences in the amounts obtained from TG and isotherm data can derive from different factors, such as the contribution of co-adsorbed water molecules to TG results. In addition, saturation capacity is a limit value estimated from non-linear fitting of experimental data (see Figure 1) and it does not refer to a single adsorption measurement.

**Figure 3:**
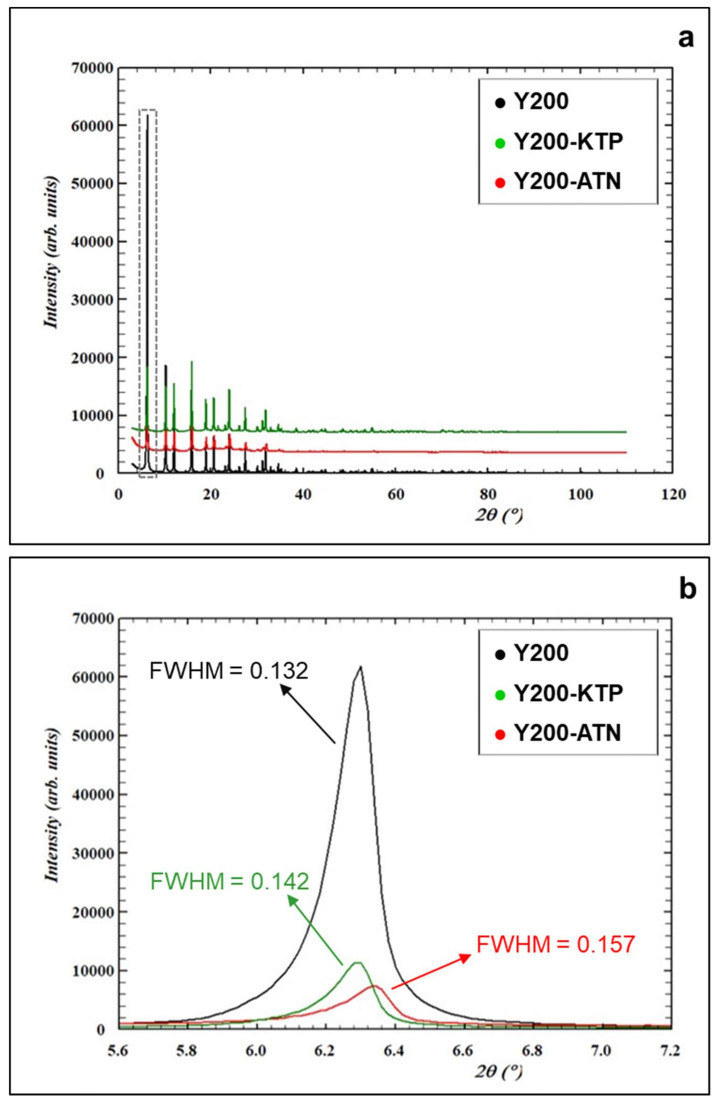
X-ray powders diffraction pattern in the entire 2ϑ range investigated (**a**) and in the low 2ϑ region (**b**) of as-synthesized Y200 (black), Y200 saturated with KTP (green), and ATN (red).

The Y200 dealuminated zeolite is characterized by the presence of both microporosity and macroporosity mainly due to the dealumination process [39]. Although drug adsorption can occur in both micro- and mesoporous structures, the deeply investigation of the adsorption site location lies beyond the aim of the present work. In order to have an experimental evidence of the effective incorporation of the drug inside the zeolite porosity and not only on the zeolite surface, a structural investigation was carried out. Figure 3 shows the results attained for Y200-KTP and Y200-ATN systems. Structural analyses carried out on Y200 saturated with HCT led to results similar to those obtained for KTP: For this reason, the diffractometric data of HCT have not been reported here.

The XRD diffraction patterns of Y200 before and after KTP and ATN adsorption showed changes in the entire 2ϑ range investigated (Figure 3a) and especially in the low 2ϑ region (Figure 3b). After drugs adsorption, the intensity of the (111) reflection decreased and the full width at half maximum (FWHM), reported in Figure 3b, increased, thus indicating changes in crystallinity of the original sample. At the same time, shifts in the peaks positions revealed unit cell parameters modifications (Table 2) whereas variations in their intensity suggested modifications induced by KTP and ATN adsorption, indicating that the molecules are effectively penetrated inside the structural microporosities of Y200.

Considering the structural difference between the channels, the framework oxygens distances between the pores give important indications in determining the stability of a sorption site. A guest molecule on a stable sorption site favors those distances that minimize the van der Waals interaction potentials. This means that due to the host-guest interactions, the pore sizes of the channels are roughly the same, but the precise structures of the channels are quite different. The bond lengths and angles in the framework geometry and the diagonal O—O internuclear distances in the 12-ring channel (12MR) are listed in Table 2. The 12MR diameters, calculated from the Rietveld structure refinement, are indicated by O1-O1 and O4-O4 distances, treating the framework oxygen atoms as cold hard spheres with van der Waals radius equal to 1.35 Å. The oblate spheroidal KTP and ATN profile “forced” the 12-ring channel to adapt to the host molecules geometry, with a consequent variation of Crystallographic Free Area (CFA). The 12MR had been compressed becoming more elliptical, as expressed by the channel ellipticity (ε), defined as the ratio between the smaller and the larger O–O “free diameters” of the 12-rings. The refined atomic occupancy factors gave rise to an adsorption of 19% and 18% in weight for KTP and ATN, respectively. This finding is in good agreement with those determined by adsorption isotherms (see q_S_ in Table 1) and by thermogravimetric analyses (see Figure 2). The positions occupied by atenolol and ketoprofen atoms appeared strongly disordered due to real static disorder or to dynamic disorder. For these reasons, the complete geometry of selected molecules inside the supercage was not achieved. The benzene ring of the drugs molecule was recognized in the supercage (Figure S1 in Supporting Information) and the adsorption site was partially occupied in both cases and each shows, on a statistical basis, four possible orientations. In particular, two and four orientations were identified for the tert-butyl and methoxyl groups, respectively. This result could indicate that structural and topological information contents may be significantly different, due to the geometry of extra-framework species, their ordering and respective symmetry lowering, framework distortions, etc. Consequently, we cannot exclude that the FAU framework could be in higher symmetry (F d -3) compared to the symmetry of the guest molecules. Moreover, the absence of cubic forbidden peaks indicating change in the space group does not get reported after adsorption, consequently, the F d -3 one was also adopted in the structure refinement.

**Figure 4:**
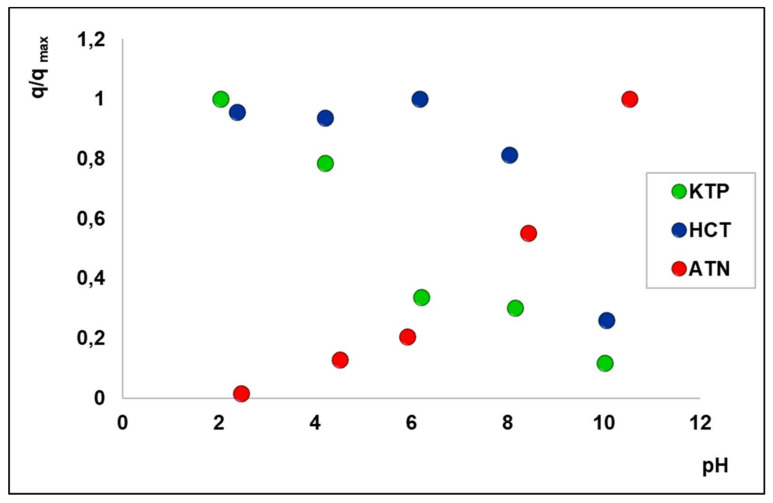
Normalized adsorbed amount (q/q_max_) on Y200 of KTP (green), HCT (blue), and ATN (red) vs. pH.

Since it is known that the solution pH influences the adsorption of molecules with ionizable functional groups [40], adsorption experiments were carried out in water at different pH. The pH-dependent adsorption behaviour was studied by measuring the amount of adsorbed drug (q), normalized to the maximum value found in the investigated pH range (q_max_) for each analyte vs. the pH of the solution (see Figure 4). The pK_a_ values of analytes are reported in Supplementary Materials (Table S2).

At 2 < pH < 12, the zeolite surface remains negatively charged due to the dissociation of the hydroxyl groups [41]. In the explored pH range, literature data [42] reported that the Y zeolite does not undergo degradation.

The pK_a_ of KTP is 4.0, hence at pH < 4 KTP is mainly in its neutral form and at pH > 4 it is negatively charged. As shown in Figure 4, the adsorption of KTP decreases as pH increases, due to repulsive electrostatic interactions between KTP and the zeolite surface, both negatively charged. Analogously to KTP, the molecule of HCT (pK_a1_ = 7.9) is undissociated at pH < pK_a1_ and negatively charged in more alkaline solutions. The adsorption is almost constant until the HCT molecule remains neutral and decreases as the negatively charged molecule becomes dominant. These results demonstrated that KTP and HCT interact with the high-silica zeolite mainly in their neutral form thanks to hydrophobic interactions. The preferential adsorption of neutral organic micropollutants by high-silica zeolites was observed also by Fukahori et al. [40] who reported that sulfa-drugs in neutral form could be more readily adsorbed onto FAU zeolites than those in cationic and anionic forms, based on the hydrophobic interactions. On the contrary, when the anionic forms are dominant under alkaline conditions, a reduction of adsorption capacity occurs [31]. Atenolol showed an opposite behaviour with respect to other two drugs, likewise to what was observed for BEA zeolites [15]. It should be minded that this molecule is cationic at pH < pK_a_ and neutral at pH > pK_a_ (pK_a_ = 9.6). As the pH increases, the conversion of cationic species in the neutral one may result in greater hydrophobic interactions with Y200 and, at the same time, a lower solubility of the neutral ATN [43] which displays higher affinity with the sorbent surface rather than the liquid phase. Both these aspects could be responsible of the increase of ATN adsorption with pH.

### 2.2. Release

The performances of Y200 as a pre-concentration media for drugs enrichment was compared to a BEA zeolite having SAR 25, which was previously calcined (referred to as Beta25c), since it has been proved that this thermal treatment increases the adsorption efficiency of BEAs, thanks to the increase of acidity due to the thermal release of ammonia for NH_4_-zeolites and to the increase of acidity as well as to surface and structural modifications induced by thermal treatments [15,18]. The extracting phases, at different pH and organic modifier contents, were selected in order to minimize the attractive interactions between the sorbed drug and the zeolite framework (see Figure 4) and, at the same time, to favour the analyte’s dissolution in the liquid phase. In general, for each zeolite-drug system, the following extracting phases were tested: Water, an organic polar solvent (methanol and acetonitrile), aqueous solutions at pH values where the adsorption has demonstrated to be low, mixtures organic modifier/formic acid or organic modifier/ammonium hydroxide. Despite the fact that in some cases acidic or alkaline extracting phases were employed, the time of contact of 5 min with the loaded zeolite (see Section 3.3) is too brief to induce structural degradation. Moreover, it should be considered that, in the studied pH range, it has been reported that zeolites [42,44] do not undergo degradation.

The release efficiency was calculated as a percentage recovery, according to Equation (2) [45]:

(2) $$\%R=100\;\frac{C_{f}\;V_{f}}{C_{i}\;V_{i}}$$

where C_f_ corresponds to the concentration (mg L^−1^) of the released drug in the final volume V_f_ (0.2 · 10^−3^ L), V_i_ is the initial volume (1 · 10^−3^ L), C_i_ is the initial drug concentration (in the range 0.05–1 mg L^−1^). The details of the dispersive-SPE procedure were reported in Section 3.3. In Table 3, the results obtained from dispersive-SPE applied to KTP, at an initial concentration of 1 mg L^−1^, are reported.

For both zeolites, KTP is not at all released in water and the release is still negligible even by increasing the pH of water to 10. At this pH, KTP is negatively charged, hence electrostatic repulsions with a zeolite framework, negatively charged too, were expected. Percent recoveries higher than 80% were obtained with mixtures of methanol and formic acid, probably due to interactions between the neutral form of KTP at pH < pK_a_ and the extracting phase enriched in an organic modifier.

**Figure 5:**
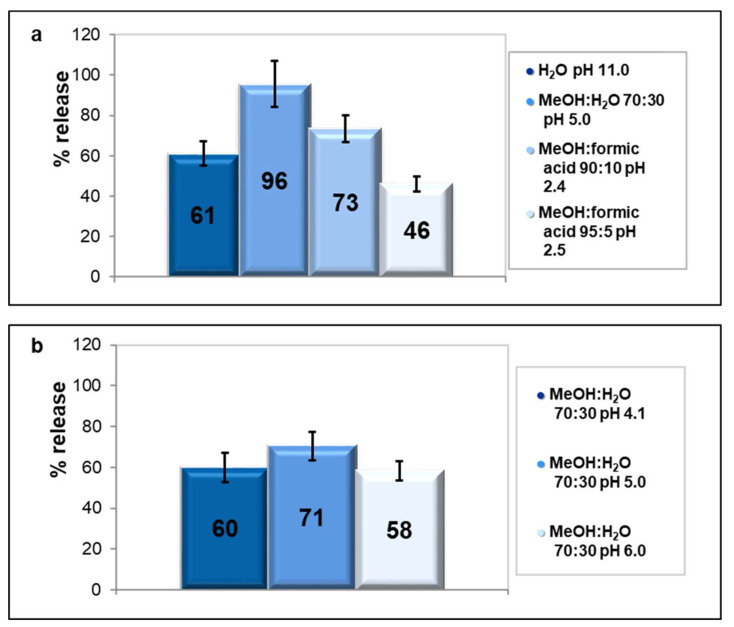
Release of HCT 1 mg L^−1^ from Y200 with different extracting phases (**a**) and from Beta25c with mixture MeOH:H_2_ O 70:30 at different pH (**b**).

Some further experiments were carried out by varying the initial drug’s concentration, in order to verify if such positive results could be achieved also in the low concentrations range. The data reported in Supplementary Materials (Figure S2) confirm that great recovery efficiencies were achieved with both zeolites also at low analyte concentrations (1, 0.1, and 0.05 mg L^−1^). The results of the present work were compared with those obtained by other studies [8,46] focused on solid phase extraction of KTP by using a commercial adsorbent and the efficiency was found comparable. Figure 5 shows some of the results obtained by extraction of HCT with different phases from both zeolites.

The release of HCT in water from Y200 is low but not negligible. However, a higher release (61 ± 6% as shown in Figure 5a) was obtained by increasing the pH to 10, because of electrostatic repulsions between the negatively charged drug and the zeolitic framework. A high recovery of 96 ± 11% was obtained with MeOH:H_2_ O = 70:30 at pH 5.0: analogously to KTP, in these conditions (pH < pK_a_) HCT is neutral and displays high affinity with the extracting phase which is mainly organic. Some experiments were carried out also at HCT concentrations lower than 1 mg L^−1^ (i.e., 0.1 and 0.05 mg L^−1^): No significant differences were observed between the different concentrations, thus indicating that the dispersive SPE of the Y200-HCT system is effective also in the low concentrations range, similarly for what was observed for KTP. The recovery efficiency on Beta25c (Figure 5b) was evaluated by keeping constant the composition of the extracting phase and by varying the pH from 4 to 6. The highest recovery (71 ± 7%) was obtained at pH 5 as already found for Y200, even if the extraction was less efficient. This could be due to higher affinities between HCT at low concentrations and Beta25c (a binding constant of 0.60 L mg^−1^ was found [15]) with respect to Y200 (b = 0.037 L mg^−1^ as reported in Table 1). Finally, for that which concerns ATN, this drug was not at all released from Y200 in water at pH 3, despite the fact that it was found that the adsorption is negligible at strongly acidic pH (Figure 4). Other experiments were performed at acidic pH by adding an organic modifier, and the best result (71 ± 5%) was obtained with a mixture (ACN:formic acid) (95:5) as an extracting phase. Further tests were carried out at high pH values, by using extracting phases constituted by an organic modifier (MeOH, ACN, or a mixture of them) and ammonium hydroxide at different concentrations. These alkaline conditions should promote the dissolution of neutral ATN into the liquid phase which is mainly organic. The highest efficiency (83 ± 9%) was obtained with an extracting phase composed by (MeOH:ACN 40:60):(NH_4_ OH 5%) = 70:30. The solute enrichment obtained with the proposed procedure (five-fold enrichment of concentration, as described in Section 3.3) could be improved by using higher volumes of the initial drug solution. Moreover, the extraction efficiency could be enhanced by using longer time contact or higher volume of extraction phases. However, the results herein presented could be considered a good compromise between the efficiency of the enrichment process (> 80%) and the cost and time saving of the concentration procedure. Unfortunately, for the Beta25c-ATN system, very low recoveries were obtained with several extracting phases, in fact the best result was reached with a mixture (MeOH:ACN 40:60):(NH_4_ OH 5%) = 80:20 which led to a scarce 13 ± 2%. The differences in terms of recovery efficiencies between the two selected zeolites could be ascribed to the binding constants. The b parameter of the Beta25c-ATN system was found to be 5.8 ± 2.8 L mg^−1^ [15], while that of the Y200-ATN system was 0.11 ± 0.03 L mg^−1^ (Table 1). As already hypothesized for HCT and KTP, the lower recovery of ATN from Beta25c could be due to its higher affinity with respect to Y200.

## 3. Materials and Methods

### 3.1. Materials

Technical grade (99% purity) ketoprofen (KTP), hydrochlorothiazide (HCT), and atenolol (ATN) were obtained from Sigma-Aldrich (Steinheim, Germany), as well as sodium phosphate monobasic for the HPLC eluent. HPLC grade acetonitrile (ACN) and methanol (MeOH) were purchased from VWR International PBI Srl (Radnor, PA, USA). The water was Milli-Q grade (Merck Millipore, Burlington, MA, USA). Formic acid and ammonium hydroxide solution were obtained from Merck (Darmstadt, Germany). Hydrochloric acid and sodium hydroxide (Alfa Aesar, Haverhill, MA, USA) were used for pH adjustment. The pH was measured with an Amel 2335 pH-meter (Milano, Italy). Zeolite powders were obtained from Zeolyst International (Conshohocken, PA, USA) and from Tosoh Corporation (Tokyo, Japan). The main features of the selected zeolites are reported in Supplementary Materials (Table S1). In our study, two USY zeolites with a Silica/Alumina Ratio (SAR) of 30 and 200 (referred as Y30 and Y200) and a BEA with SAR of 25 (referred as Beta25) have been employed. According to Čejka et al. [29], who discriminated between high-silica and low-silica zeolites when SAR was respectively above or below 5, the selected materials can be considered high-silica zeolites. Structures and chemical properties of the selected drugs are reported in Supplementary Materials (Table S2) [47,48,49,50].

### 3.2. Sorption Studies

The adsorption experiments were carried out using the batch method, by putting in contact aqueous solutions of drugs at different concentrations in Milli-Q water with a known amount of zeolite. It has been reported in previous studies [15] that the equilibrium is quite fast, nevertheless in this work a contact time of 24 h was employed. Batch experiments were carried out in duplicate in 25 mL crimp top reaction glass flasks sealed with PTFE septa (Supelco, Bellefonte, PA, USA). During the equilibration, the solutions were thermostated at a temperature of 25.3 ± 0.5 °C and continuously stirred at 700 rpm. The solid was separated from the solution by centrifugation (Eppendorf 5418, Hamburg, Germany) at 14,000 rpm for 10 min. For each level of concentration, the amount of analyte in the solution, before and after the contact with the adsorbent, was determined by HPLC-DAD as described in Supporting Information. Thermogravimetric and diffractometric analyses were performed on zeolites before and after saturation with the selected drugs. Thermogravimetric (TG) measurements were performed in air up to 900 °C, at 10 °C min^−1^ heating rate, using an STA 409 PC LUXX^®^ - Netzch Gerätebau GmbH (Verona, Italy). The X-ray diffraction analysis was carried out on a Bruker D8 Advance (Karlsruhe, Germany) diffractometer (Cu Kα1,2 radiation) equipped with a Si (Li)SOL-X solid-state detector. The GSAS software and the EXPGUI graphical interface were employed for structural refinements through a full profile Rietveld analysis. Statistical elaborations were carried out through MATLAB^®^ ver. 9.1 software (The MathWorks Inc., Natick, MA, USA).

### 3.3. Dispersive-SPE Studies

The dispersive-SPE procedure was carried out as follows: In a safe-lock microcentrifuge tube, 2 mg of zeolite were added to 1 mL of drug solution, in a concentration range of 0.05–1 mg L^−1^ in Milli-Q water, and mixed for 5 min by vortex (VWR International PBI Srl, Radnor, PA, USA) equipped with a specific adapter for microcentrifuge tubes. The resulting suspension was centrifuged at 14,000 rpm for 5 min and the supernatant was analyzed by HPLC-DAD, in order to quantify the adsorption. In all cases, it was found that the drug concentration in the supernatant was below the instrumental limit of detection. Therefore, the drug was completely adsorbed in the brief time of contact, thanks to the fast kinetic offered by the zeolites [14,15], especially in the low concentration range. After the supernatant’s removal, the loaded zeolite was re-suspended for 5 min with 500 μL of solutions having different compositions (i.e., pH and organic modifier concentration) and then centrifuged at 14,000 rpm for 5 min. Finally, the supernatant was dried by evaporation under a gentle stream of nitrogen gas, dissolved with 200 μL of the HPLC mobile phase (see Supplementary Materials) and analyzed by HPLC-DAD for the quantification of the released drug. With the proposed procedure, a five-fold enrichment was achieved. All measurements were performed in triplicate.

## 4. Conclusions

In this study, the adsorptive properties of two commercial high-silica Y zeolites, with a Silica/Alumina Ratio 30 and 200 (namely Y30 and Y200), toward drugs in a diluted aqueous solution were evaluated, with the aim to investigate their possible use in the enrichment step of the analysis. In fact, the determination of pharmaceuticals at trace levels in natural waters requires advanced analytical methodologies enabling a more efficient detection with lower limits of quantitation. Owing to their high surface area, hydrophobicity and water stability, the selected materials were demonstrated to be efficient sorbents, both in terms of saturation capacity and binding constant. Among the two Y-zeolites, in general better performances were observed for Y200. The structural analyses of saturated zeolites revealed that drugs adsorption occurred inside the porosities of Y200 leading to modifications of lattice parameters and channel ellipticity. Pharmaceuticals pre-concentration from dilute aqueous solutions was carried out through the dispersive-solid phase extraction technique, thanks to its attractive features, such as the short experimental times and small reagents amount required. As a pre-concentration medium, in addition to Y200, a calcined Beta-zeolite with Silica/Alumina Ratio 25 was tested. The dispersive-solid phase extraction experiments led to high recoveries, especially for ketoprofen and hydrochlorothiazide. These results are promising, especially by considering the short operational times required for the entire procedure.

## Figures and Tables

**Table 1:** Parameters Estimated by Non-Linear Fitting, According to the Langmuir Model, of Three Drugs Adsorption on Y200 and Y30. The Confidence Limits at 95% of Probability of Parameters are Reported in Brackets.

Drug-Zeolite	b (L mg ^−1^)	q_S_ (mg g ^−1^)	R^2^
KTP–Y200	0.91 (0.76; 1.1)	208 (202; 214)	0.9887
HCT–Y200	0.037 (0.029; 0.044)	322 (302; 342)	0.9856
ATN–Y200	0.11 (0.082; 0.14)	183 (171; 195)	0.9737
KTP–Y30	0.052 (0.035; 0.069)	173 (148; 197)	0.9756
HCT–Y30	0.022 (0.018; 0.026)	121 (114; 128)	0.9957
ATN–Y30	0.084 (0.058; 0.11)	240 (223; 257)	0.9727

**Table 2:** Lattice Parameters for Y200 Before and After Pharmaceuticals Adsorption.

	Y200	Y200-KTP	Y200-ATN
Crystallite size (Å)	668.5	621.5	563.4
a = b = c (Å)	24.259(1)	24.255(1)	24.159(1)
V (Å^3^)	14,277.1(1)	14,269.1(1)	14,101.5(1)
O4-O4 (Å)	9.81	10.30	9.90
O1-O1 (Å)	9.70	9.63	9.58
C.F.A. (Å^2^)	39.07	41.43	38.90
Ellipticity (ε)	1.01	1.07	1.03

**Table 3:** Percentage Release of KTP at an Initial Concentration of 1 mg L^−1^ from Y200 and Beta25c (± SD: Standard Deviation).

Extracting Phase	Y200	Beta25c
ACN	53 ± 4	53 ± 5
MeOH	63 ± 8	47 ± 3
MeOH:formic acid 90:10 pH 2.4	94 ± 14	115 ± 17
MeOH:formic acid 95:5 pH 2.5	88 ± 11	86 ± 12

## Supplementary Materials

The following are available online at; Table S1: Zeolites characteristics; Table S2: Molecular structures and physical chemical properties of the studied drugs; Section S1: Experimental HPLC/DAD; Figure S1: Adsorption site in the supercage for ATN and KTP benzene ring; Figure S2: Release of KTP at three concentration levels with MeOH:formic acid 90:10 pH 2.4 as an extracting phase.

## References

[1] Charuaud L., Jarde E., Jaffrezic A., Thomas M., le Bot B. (2019). Veterinary pharmaceutical residues from natural water to tap water: Sales, occurrence and fate. J. Hazard. Mater..

[2] Sharma B.M., Bečanová J., Scheringer M., Sharma A., Bharat G.K., Whitehead P.G., Klánová J., Nizzetto L. (2019). Health and ecological risk assessment of emerging contaminants (pharmaceuticals, personal care products, and artificial sweeteners) in surface and groundwater (drinking water) in the Ganges River Basin, India. Sci. Total Environ..

[3] Yang Y., Ok Y.S., Kim K.H., Kwon E.E., Tsang Y.F. (2017). Occurrences and removal of pharmaceuticals and personal care products (PPCPs) in drinking water and water/sewage treatment plants: A review. Sci. Total Environ..

[4] McCallum E.S., Krutzelmann E., Brodin T., Fick J., Sundelin A., Balshine S. (2017). Exposure to wastewater effluent affects fish behaviour and tissue-specific uptake of pharmaceuticals. Sci. Total Environ..

[5] Wilkinson J., Hooda P.S., Barker J., Barton S., Swinden J. (2017). Occurrence, fate and transformation of emerging contaminants in water: An overarching review of the field. Environ. Pollut..

[6] Yang H., Lu G., Yan Z., Liu J., Dong H., Jiang R., Zhou R., Zhang P., Sun Y., Nkoom M. (2019). Occurrence, spatial-temporal distribution and ecological risks of pharmaceuticals and personal care products response to water diversion across the rivers in Nanjing, China. Environ. Pollut..

[7] Furlong E.T., Batt A.L., Glassmeyer S.T., Noriega M.C., Kolpin D.W., Mash H., Schenck K.M. (2017). Nationwide reconnaissance of contaminants of emerging concern in source and treated drinking waters of the United States: Pharmaceuticals. Sci. Total Environ..

[8] Caban M., Lis E., Kumirska J., Stepnowski P. (2015). Determination of pharmaceutical residues in drinking water in Poland using a new SPE-GC-MS(SIM) method based on Speedisk extraction disks and DIMETRIS derivatization. Sci. Total Environ..

[9] Comber S., Gardner M., Sörme P., Leverett D., Ellord B. (2018). Active pharmaceutical ingredients entering the aquatic environment from wastewater treatment works: A cause for concern?. Sci. Total Environ..

[10] Patrolecco L., Capri S., Ademollo N. (2015). Occurrence of selected pharmaceuticals in the principal sewage treatment plants in Rome (Italy) and in the receiving surface waters. Environ. Sci. Pollut. Res..

[11] Carmalin S.A., Eder C.L. (2018). Removal of emerging contaminants from the environment by adsorption. Ecotox. Environ. Safe.

[12] Patiño Y., Díaz E., Ordóñez S. (2016). Pre-concentration of nalidixic acid through adsorption–desorption cycles: Adsorbent selection and modelling. Chem. Eng. J..

[13] Naing N.N., Li S.F.Y., Lee H.K. (2016). Evaluation of graphene-based sorbent in the determination of polar environmental contaminants in water by micro-solid phase extraction-high performance liquid chromatography. J. Chromatogr. A.

[14] Martucci A., Pasti L., Marchetti N., Cavazzini A., Dondi F., Alberti A. (2012). Adsorption of pharmaceuticals from aqueous solutions on synthetic zeolites. Micropor. Mesopor. Mat..

[15] Pasti L., Sarti E., Cavazzini A., Marchetti N., Dondi F., Martucci A. (2013). Factors affecting drug adsorption on beta zeolites. J. Sep. Sci..

[16] Costa A.A., Wilson W.B., Wang H., Campiglia A.D., Dias J.A., Dias S.C.L. (2012). Comparison of BEA, USY and ZSM-5 for the quantitative extraction of polycyclic aromatic hydrocarbons from water samples. Micropor. Mesopor. Mat..

[17] Wilson W.B., Costa A.A., Wang H., Campiglia A.D., Dias J.A., Dias S.C.L. (2013). Pre-concentration of water samples with BEA zeolite for the direct determination of polycyclic aromatic hydrocarbons with laser-excited time-resolved Shpol’skii spectroscopy. Microchem. J..

[18] Sarti E., Chenet T., Pasti L., Cavazzini A., Rodeghero E., Martucci A. (2017). Effect of silica alumina ratio and thermal treatment of Beta zeolites on the adsorption of toluene from aqueous solutions. Minerals.

[19] Martucci A., Braschi I., Bisio C., Sarti E., Rodeghero E., Bagatin R., Pasti L. (2015). Influence of water on the retention of methyl tertiary-butyl ether by high silica ZSM-5 and Y zeolites: A multidisciplinary study on the adsorption from liquid and gas phase. RSC Adv..

[20] Pasti L., Rodeghero E., Beltrami G., Ardit M., Sarti E., Chenet T., Stevanin C., Martucci A. (2018). Insights into adsorption of chlorobenzene in high silica MFI and FAU zeolites gained from chromatographic and diffractometric techniques. Minerals.

[21] Anastassiades M., Lehotay S.J., Stajnbaher D., Schenck F.J. (2003). Fast and easy multiresidue method employing acetonitrile extraction/partitioning and “dispersive solid-phase extraction” for the determination of pesticide residues in produce. J. AOAC Int..

[22] Román I.P., Chisvert A., Canals A. (2011). Dispersive solid-phase extraction based on oleic acid-coated magnetic nanoparticles followed by gas chromatography–mass spectrometry for UV-filter determination in water samples. J. Chromatogr. A.

[23] Wang P., Yang X., Wang J., Cui J., Dong A.J., Zhao H.T., Zhang L.W., Wang Z.Y., Xu R.B., Li W.J. (2012). Multi-residue method for determination of seven neonicotinoid insecticides in grains using dispersive solid-phase extraction and dispersive liquid–liquid micro-extraction by high performance liquid chromatography. Food Chem..

[24] Tsai W.H., Huang T.C., Huang J.J., Hsue Y.H., Chuang H.Y. (2009). Dispersive solid-phase microextraction method for sample extraction in the analysis of four tetracyclines in water and milk samples by high-performance liquid chromatography with diode-array detection. J. Chromatogr. A..

[25] Rodeghero E., Martucci A., Cruciani G., Bagatin R., Sarti E., Bosi V., Pasti L. (2016). Kinetics and dynamic behaviour of toluene desorption from ZSM-5using in situ high-temperature synchrotron powder X-ray diffraction and chromatographic techniques. Catal. Today.

[26] Rodeghero E., Pasti L., Sarti E., Cruciani G., Bagatin R., Martucci A. (2017). Temperature-induced desorption of methyl tert-butyl ether confined on ZSM-5: An in situ synchrotron XRD powder diffraction study. Minerals.

[27] Archer E., Petrie B., Kasprzyk-Hordern B., Wolfaard G.M. (2017). The fate of pharmaceuticals and personal care products (PPCPs), endocrine disrupting contaminants (EDCs), metabolites and illicit drugs in a WWTW and environmental waters. Chemosphere.

[28] Fernández-Perales M., Sánchez-Polo M., Rozalen M., López-Ramón M.V., Mota A.J., Rivera-Utrilla J. (2020). Degradation of the diuretic hydrochlorothiazide by UV/Solar radiation assisted oxidation processes. J. Environ. Manag..

[29] Čejka J., van Bekkum H., Corma A., Schüth F. (2007). Introduction to Zeolite Science and Practice.

[30] Hartig D., Schwindt N., Scholl S. (2017). Using the local adsorption equilibrium distribution based on a Langmuir type adsorption model to investigate liquid phase adsorption of sugars on zeolite BEA. Adsorption.

[31] Jiang N., Shang R., Heijman S.G.J., Rietveld L.C. (2018). High-silica zeolites for adsorption of organic micro-pollutants in water treatment: A review. Water Res..

[32] Cuerda-Correa E.M., Domínguez-Vargas J.R., Olivares-Marín F.J., Beltrán de Heredia J. (2010). On the use of carbon blacks as potential low-cost adsorbents for the removal of non-steroidal anti-inflammatory drugs from river water. J. Hazard. Mater..

[33] Haro N.K., del Vecchio P., Marcilio N.R., Féris L.A. (2017). Removal of atenolol by adsorption – Study of kinetics and equilibrium. J. Clean. Prod..

[34] Fröhlich A.C., Foletto E.L., Dotto G.L. (2019). Preparation and characterization of NiFe_2_ O_4_/activated carbon composite as potential magnetic adsorbent for removal of ibuprofen and ketoprofen pharmaceuticals from aqueous solutions. J. Clean. Prod..

[35] Arya V., Philip L. (2016). Adsorption of pharmaceuticals in water using Fe_3_ O_4_ coated polymer clay composite. Micropor. Mesopor. Mat..

[36] Wesolowski M., Rojek B. (2013). Thermogravimetric detection of incompatibilities between atenolol and excipients using multivariate techniques. J. Therm. Anal. Calorim..

[37] Silva Pires M.A., Souza dos Santos R.A., Sinisterra R.D. (2011). Pharmaceutical composition of hydrochlorothiazide: β-cyclodextrin: Preparation by three different methods, physico-chemical characterization and in vivo diuretic activity evaluation. Molecules.

[38] Tiţa B., Fuliaş A., Bandur G., Marian E., Tiţa D. (2011). Compatibility study between ketoprofen and pharmaceutical excipients used in solid dosage forms. J. Pharm. Biomed..

[39] Braschi I., Gatti G., Bisio C., Berlier G., Sacchetto V., Cossi M., Marchese L. (2012). The role of silanols in the interactions between methyl tert-butyl ether and high-silica Faujasite Y: An infrared spectroscopy and computational model study. J. Phys. Chem. C.

[40] Fukahori S., Fujiwara T., Ito R., Funamizu N. (2011). pH-Dependent adsorption of sulfa drugs on high silica zeolite: Modeling and kinetic study. Desalination.

[41] Kuzniatsova T., Kim Y., Shqau K., Dutta P.K., Verweij H. (2007). Zeta potential measurements of zeolite Y: Application in homogeneous deposition of particle coatings. Micropor. Mesopor. Mat..

[42] Lee E.F.T., Rees L.V.C. (1987). Dealumination of sodium Y zeolite with hydrochloric acid. J. Chem. Soc. Faraday Trans..

[43] Schönherr D., Wollatz U., Haznar-Garbacz D., Hanke U., Box K.J., Taylor R., Ruiz R., Beato S., Becker D., Weitschies W. (2015). Characterisation of selected active agents regarding pKa values, solubility concentrations and pH profiles by Sirius T3. Eur. J. Pharm. Biopharm..

[44] Liu X., Mäki-Arvela P., Aho A., Vajglova Z., Gun’ko V.M., Heinmaa I., Kumar N., Murzin D.Y. (2018). Zeta potential of beta zeolites: Influence of structure, acidity, pH, temperature and concentration. Molecules.

[45] Bosi V., Sarti E., Navacchia M.L., Perrone D., Pasti L., Cavazzini A., Capobianco M.L. (2015). Gold-nanoparticle extractioned reversed-electrode-polarity stacking mode combined to enhance capillary electrophoresis sensitivity for conjugated nucleosides and oligonucleotides containing thioether linkers. Anal. Bioanal. Chem..

[46] Santos J.L., Aparicio I., Alonso E., Callejón M. (2005). Simultaneous determination of pharmaceutically active compounds in wastewater samples by solid phase extraction and high-performance liquid chromatography with diode array and fluorescence detectors. Anal. Chim. Acta.

[47] Gantiva M., Martínez F. (2010). Thermodynamic analysis of the solubility of ketoprofen in some propylene glycol + water cosolvent mixtures. Fluid Phase Equilib..

[48] Radjenović J., Petrović M., Ventura F., Barceló D. (2008). Rejection of pharmaceuticals in nanofiltration and reverse osmosis membrane drinking water treatment. Water Res..

[49] Kadam Y., Yerramilli U., Bahadur A., Bahadur P. (2011). Micelles from PEO–PPO–PEO block copolymers as nanocontainers for solubilization of a poorly water soluble drug hydrochlorothiazide. Colloid. Surface B.

[50] Küster A., Alder A.C., Escher B.I.K., Duis K., Fenner K., Garric J., Hutchinson T.H., Lapen D.R., Péry A., Rö#xF6;mbke J. (2010). Environmental risk assessment of human pharmaceuticals in the European Union: A case study with the β-blocker atenolol. Integr. Environ. Assess. Manag..

