# Supplementary material for: High-Silica Zeolites as Sorbent Media for Adsorption and Pre-Concentration of Pharmaceuticals in Aqueous Solutions
Source: Molecules. 2020 Jul 22;25(15):3331. doi: 10.3390/molecules25153331 (PMC7436148; doi:10.3390/molecules25153331)
Supplement: Supplementary file 1 [file molecules-25-03331-s001.pdf]

SUPPORTING INFORMATION

High-Silica Zeolites as Sorbent Media for Adsorption and Pre-Concentration of Pharmaceuticals in Aqueous Solutions

Elena Sarti [1,*], Tatiana Chenet [1], Claudia Stevanin [1], Valentina Costa [1], Alberto Cavazzini [1], Martina Catani [1], Annalisa Martucci [2], Nicola Precisvalle [2], Giada Beltrami [2] and Luisa Pasti [1]

[1] Department of Chemical and Pharmaceutical Sciences, University of Ferrara, Via Luigi Borsari 46, 44121 Ferrara, Italy; tatiana.chenet@unife.it (T.C.); claudia.stevanin@unife.it (C.S.); valentina.costa@unife.it (V.C.), alberto.cavazzini@unife.it (A.C.); martina.catani@unife.it (M.C.); luisa.pasti@unife.it (L.P.)

[2] Department of Physics and Earth Sciences, University of Ferrara, Via Giuseppe Saragat 1, 44122 Ferrara, Italy; annalisa.martucci@unife.it (A.M.); nicola.precisvalle@unife.it (N.P.); giada.beltrami@unife.it (G.B.)

[*] Correspondence: elena.sarti@unife.it, Tel: +39 0532 455189; Fax: +39 0532 455507

Contains:

- Pages: 4
- Tables: 2
- Figures: 2

Contents:

Table S1. Zeolites characteristics
Table S2. Molecular structures and physical chemical properties of the studied drugs
Section S1. Experimental : HPLC/DAD
Figure S1. Adsorption site in the supercage for ATN and KTP benzene ring
Figure S2. Release of KTP at three concentration levels with MeOH:formic acid 90:10 pH 2.4 as extracting phase
References

Table S1. Zeolites characteristics

	Producer	SiO_2/Al_2O_3 ratio	Nominal cation form	Surface area ($m^2 g^{-1}$)
**Y200**	Tosoh Corporation (HSZ-390HUA)	200	Hydrogen	630
**Y30**	Zeolyst (CBV 720)	30	Hydrogen	780
**Beta25**	Zeolyst (CP814E)	25	Ammonium	680

Table S2. Molecular structures and physical chemical properties of the studied drugs

Drug	Formula	Molecular Weight	Structure	Water solubility ($g L^{-1}$)	pK_a	Log K_{ow}
**KTP**	$C_{16}H_{14}O_3$	254.28	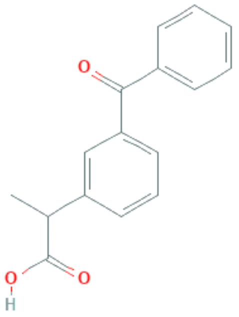	0.11 [47]	4.0 [48]	3.1 [48]
**HCT**	$C_7H_8ClN_3O_4S_2$	297.74	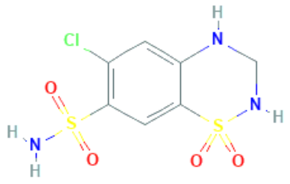	1.0 [49]	pK_{a1}=7.9 pK_{a2}=9.2 [48]	-0.07 [48]
**ATN**	$C_{14}H_{22}N_2O_3$	266.34	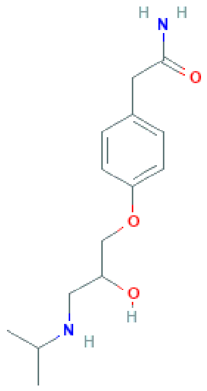	13 [50]	9.6 [50]	0.16-0.50 [50]

S1. Experimental: HPLC/DAD

Drugs solutions were analysed by a Dionex HPLC (Thermo Fisher Scientific Inc., Waltham, MA, USA) equipped with a P680 pump and a UVD340U detector. The flow rate was kept at 1 mL min^{-1} under isocratic conditions. The reversed-phase C18 analytical column (Agilent Technologies, Santa Clara, CA, USA; 150 x 4.6 mm, I.D. 5 µm) was thermostated at 25 °C. The injection volume was 20 µL. As mobile phases, the following mixtures were employed: for KTP (MeOH: phosphate buffer 2mM pH 3) (60:40), for HCT (MeOH: phosphate buffer 2mM pH 3) (15:85), for ATN (ACN: phosphate buffer 2mM pH 3) (5:95). The selected detection wavelength were 255 nm, 224 nm and 225 nm for KTP, HCT and ATN, respectively.

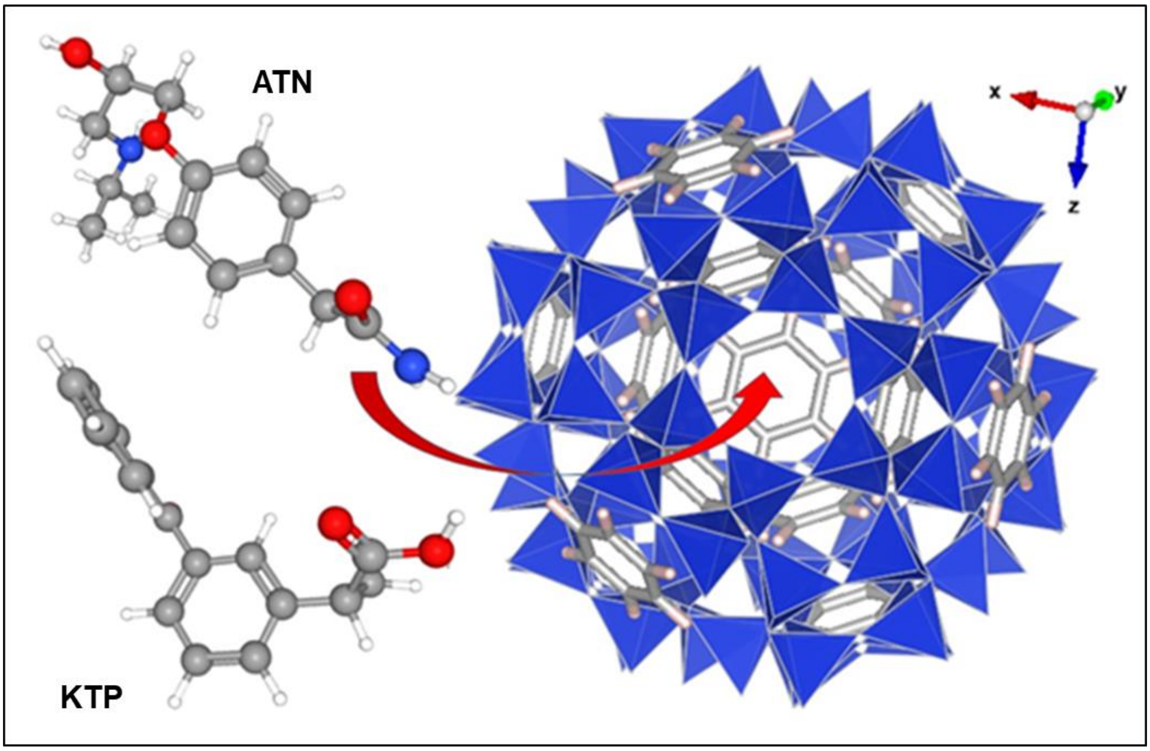

Figure S1. Adsorption site in the supercage for ATN and KTP benzene ring

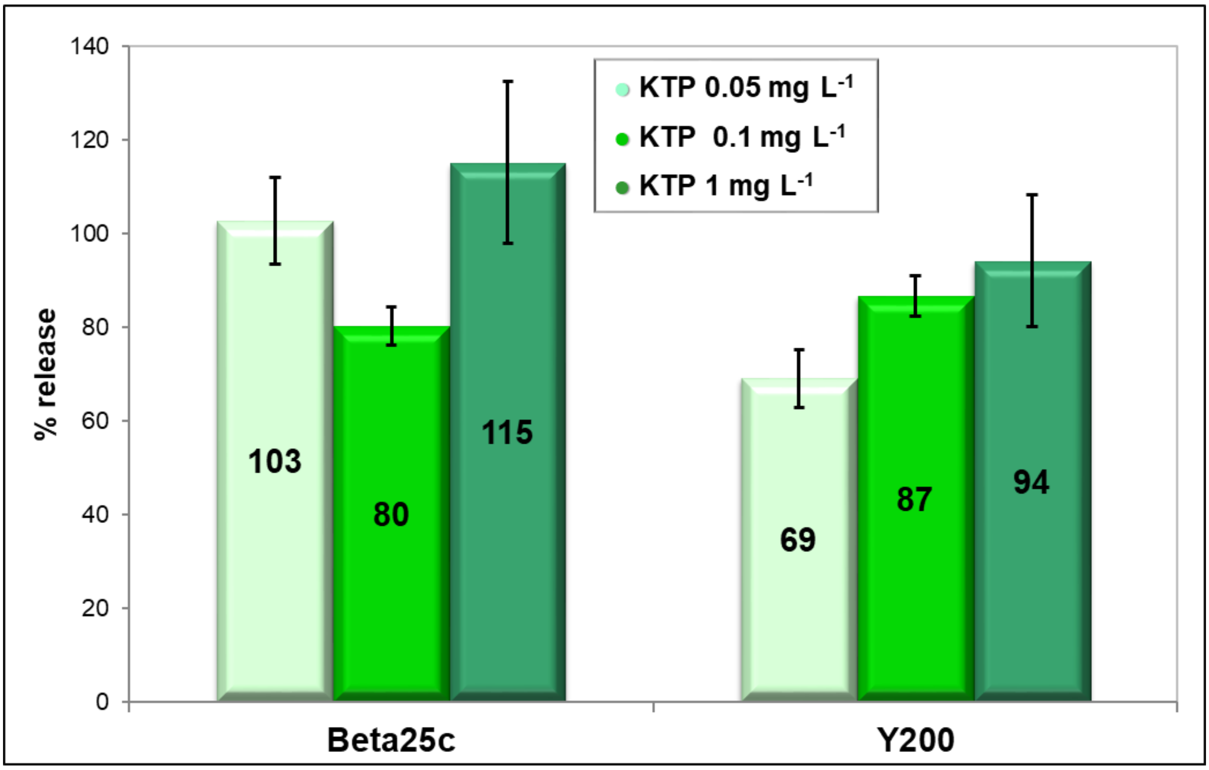

Figure S2. Release of KTP at three concentration levels with MeOH:formic acid 90:10 pH 2.4 as extracting phase

References

47. Gantiva M., Martínez F., Thermodynamic analysis of the solubility of ketoprofen in some propylene glycol + water cosolvent mixtures. *Fluid Phase Equilib.* 2010, 293, 242–250.
48. Radjenović J., Petrović M., Ventura F., Barceló D., Rejection of pharmaceuticals in nanofiltration and reverse osmosis membrane drinking water treatment. *Water Res.* 2008, 42, 3601 – 3610
49. Kadam Y., Yerramilli U., Bahadur A., Bahadur P., Micelles from PEO–PPO–PEO block copolymers as nanocontainers forsolubilization of a poorly water soluble drug hydrochlorothiazide. *Colloid. Surface B* 2011, 83, 49–57
50. Küster A., Alder A. C., Escher B. I, K. Duis K., Fenner K., Garric J., Hutchinson T. H., Lapen D. R., Péry A., Römbke J., Snape J., Ternes T., Topp E., Wehrhan A., Knacker T., Environmental risk assessment of human pharmaceuticals in the European Union: A case study with the β-blocker atenolol; *Integr. Environ. Assess. Manage.* 2010, 6, 514–523.
